# HMGB1-mediated autophagy decreases sensitivity to oxymatrine in SW982 human synovial sarcoma cells

**DOI:** 10.1038/srep37845

**Published:** 2016-11-29

**Authors:** Yongsong Cai, Peng Xu, Le Yang, Ke Xu, Jialin Zhu, Xiaoqing Wu, Congshan Jiang, Qiling Yuan, Bo Wang, Yuanbo Li, Yusheng Qiu

**Affiliations:** 1 Department of Orthopaedics of the First Affiliated Hospital, Xi’an Jiaotong University Health Science Center, Xi’an, 710061, China; 2 Department of Joint Surgery, Xi’an Hong Hui Hospital, Xi’an Jiaotong University Health Science Center, Xi’an, 710054, China; 3 Department of Biochemistry and Molecular Biology, School of Basic Medical Sciences, Xi’an Jiaotong University Health Science Center, Xi’an, 710061, China; 4 Center for Translational Medicine, the First Affiliated Hospital of Xi’an Jiaotong University Health Science Center, Xi’an, 710061, China

## Abstract

Oxymatrine (OMT) is a type of alkaloid extracted from a traditional Chinese medicinal herb, *Sophora flavescens*. Although the antitumor activities of OMT have been observed in various cancers, there are no reports regarding the effects of OMT on human synovial sarcoma. In the present study, we analyzed the antitumor activities of OMT in SW982 human synovial sarcoma cells and determine whether high mobility group box protein 1 (HMGB1)-mediated autophagy was associated with its therapeutic effects. We found that OMT exhibited antitumor activity in SW982 cells and facilitated increases in autophagy. Inhibition of autophagy by 3-MA or ATG7 siRNA increased the level of apoptosis, which indicated that OMT-induced autophagy protected cells from the cytotoxicity of OMT. Administration of OMT to SW982 cells increased the expression of HMGB1. When HMGB1 was inhibited via HMGB1-siRNA, OMT-induced autophagy was decreased, and apoptosis was increased. Furthermore, we found that HMGB1-siRNA significantly increased the expression of p-Akt and p-mTOR. OMT-induced autophagy may be mediated by the Akt/mTOR pathway, and HMGB1 plays a vital role in the regulation of autophagy. Therefore, we believe that combining OMT with an inhibitor of autophagy or HMGB1 may make OMT more effective in the treatment of human synovial sarcoma.

Synovial sarcoma (SS) is a malignant mesenchymal neoplasm that accounts for 5% to 10% of soft tissue sarcomas and occurs predominantly in adolescents and young adults at almost any anatomic site^1^. The 5-year survival rate of SS is 61–80%^2^, and the 10-year survival rate is 10–30%^3^. Approximately 70% of SSs present in the extremities, and most of these tumors metastasize to the lungs^4^. The following three histopathological subtypes exist: the biphasic type, the monophasic type and the undifferentiated type^5^. Approximately 95% of SSs are characterized by fusion between one of the *SSX* genes (*SSX1*, *SSX2*, or *SSX4*) on the X chromosome and the *SYT* gene on chromosome 18^6^. It has been confirmed that the morphological characteristics of SS are closely associated with *SYT-SSX* gene fusion^7^. The main treatment is wide surgical excision with adjuvant or neoadjuvant radiotherapy^8^. Chemotherapy has also proven helpful in achieving remission. However, drug resistance during treatment has become more common^9^,^10^. Therefore, new cytotoxic agents and novel therapeutic strategies are needed.

Oxymatrine(OMT), an alkaloid extracted from a traditional Chinese medicinal herb, *Sophora flavescens*, with a molecular formula of C15H24N2O2, has attracted much attention because of its multiple pharmacological effects and low toxicity. It has been reported that OMT exhibits a variety of pharmacological effects, including antiinflammatory^11^, anti-allergic^12^, anti-viral^13^, antifibrotic^14^, cardioprotective^15^, anti-arrhythmic^16^, analgesic^17^ and angiogenic effects^18^. More importantly, recent evidence indicates that OMT plays an important role in the treatment of various tumors, such as hepatocellular carcinoma^19^, gastric cancer^20^, colon cancer^21^, pancreatic cancer^18^, osteosarcoma^22^, prostate cancer^23^ and breast cancer^24^. However, to the best of our knowledge, there are no reports regarding the antitumor activities of OMT in human synovial sarcoma.

Autophagy is a tightly regulated lysosomal degradation pathway that is essential for cell growth, differentiation, development, survival and homeostasis^25^. It is a survival strategy employed by cells experiencing nutrient deprivation or other stresses. Many studies have shown that autophagy protects various tumor cells from apoptosis induced by chemotherapy drugs^26^,^27^. High mobility group box protein 1 (HMGB1), a highly conserved chromatin-binding nuclear protein, is very abundant and has been confirmed to play an important role in cancer development^28^,^29^,^30^. Studies have found that HMGB1 is an important regulator of autophagy and that HMGB1-mediated autophagy promotes drug resistance in tumor therapy^31^,^32^,^33^,^34^. Therefore, the aim of this study was to investigate the antitumor effects of OMT in human synovial sarcoma and determine whether HMGB1-mediated autophagy was associated with its therapeutic effects.

Based on the results of our study, we report that OMT has potent antitumor activities in SW982 human synovial sarcoma cells and that OMT-induced autophagy promotes drug resistance, which may be mediated by the HMGB1/Akt/mTOR pathway, in SW982 cells.

## Results

### OMT inhibited cell viability and induced apoptosis

**Figure 1:**
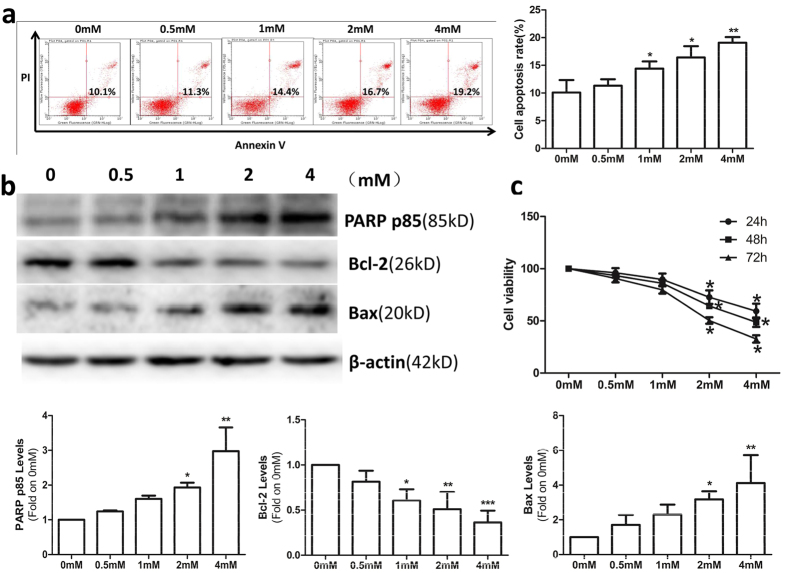
OMT inhibited cell viability and induced apoptosis. SW982 cells were treated with the indicated concentrations of OMT for 48 h. The level of apoptosis was determined with an annexin-V-FITC/PI double staining assay (**a**), and cleavage of PARP, Bcl-2 and Bax was detected by western blotting and relative densitometric analyses (**b**). Loading control was performed by evaluating β-actin expression in the same filter. Cells were treated with the indicated concentration of OMT for 24, 48 or 72 h and analyzed via MTT assay (**c**). The results are representative of 3 independent experiments and are expressed as the mean ± SD. The data were analyzed using Student’s t-test. *p < 0.05, **p < 0.01, ***p < 0.001 compared with the control (0 mM).

SW982 cells were treated with OMT for 48 h at the following concentrations: 0 mM, 0.5 mM, 1 mM, 2 mM or 4 mM. An annexin-V-FITC/PI double staining assay showed that OMT induced apoptosis in a dose-dependent manner and that the percentage of apoptotic cells was significantly increased compared to untreated cells (*p* < 0.05) (Fig. 1a). To confirm that apoptosis was induced by OMT, western blotting was performed to detect cleavage of PARP, Bcl-2 and Bax. Following OMT treatment, cleavage of PARP and the pro-apoptotic protein Bax increased, and the anti-apoptotic protein Bcl-2 decreased in a dose-dependent manner (Fig. 1b). Cell viability was detected via MTT assay. The results demonstrated that OMT inhibited cell viability in a time-and dose-dependent manner (Fig. 1c).

### OMT induced autophagy in SW982 cells

**Figure 2:**
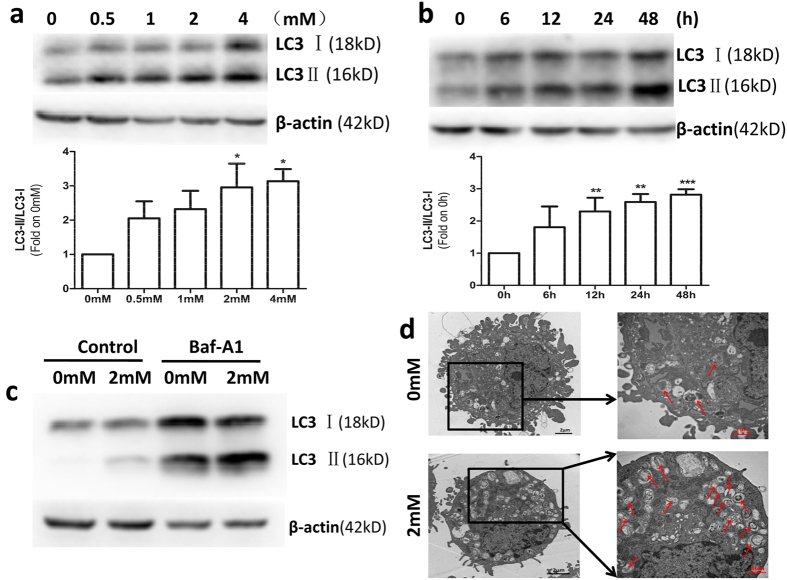
OMT induced autophagy in SW982 cells. Cells were treated with the indicated concentrations of OMT for 48 h (**a**) or 2 mM OMT for the indicated times (**b**), and the expression of LC3 was detected by western blotting and relative densitometric analyses (a and b, full-length blots are presented in Supplementary Figure S3). Autophagic flux was detected after 2 mM OMT treatment for 48 h in the presence or absence of 100 nM Baf A1 (c and Supplementary Figure S1a). Loading control was performed by evaluating β-actin expression in the same filter. After SW982 cells were treated with 2 mM OMT for 48 h, the formation of autophagosomes (→) was monitored via transmission electron microscopy (**d**). The LC3-II/LC3-I ratios from 3 independent experiments are expressed as the mean ± SD. The data were analyzed using Student’s t-test. *p < 0.05, **p < 0.01, ***p < 0.001 compared with the control (0 mM or 0 h).

It has been proven that autophagy is associated with resistance to chemotherapy in many cancer cells^35^. To investigate the effects of OMT on autophagy in SW982 cells, we detected the expression of type II LC3 (LC3-II), an autophagy marker, by western blotting. SW982 cells were treated with OMT at concentrations of 0.5 mM, 1 mM, 2 mM or 4 mM for 48 h and at a concentration of 2 mM for 6, 12, 24 or 48 h. The results showed that OMT increased the expression of LC3-II in a time- and dose-dependent manner, indicating that autophagy was induced (Fig. 2a and b).

To further investigate whether OMT regulates autophagic flux, we used two autophagic inhibitors: Baf A1, a lysosomal inhibitor, to block the autophagic pathway at a late step^36^, and E64D/pepstatin A, an inhibitor of lysosomal enzymes. The results showed that the expression of LC3-II was further increased in the presence of Baf A1 or E64d/pepstatin A via 2 mM OMT treatment in SW982 cells (Fig. 2c and Supplementary Figure S1). These results indicated that OMT treatment increased autophagic flux in SW982 cells. We also detected the expression of p62 to confirm the autophagic flux, however, no significant changes were found (Supplementary Figure S2). Furthermore, we confirmed the formation of autophagosomes by transmission electron microscopy. SW982 cells treated with 2 mM OMT for 48 h exhibited more double membrane–enclosed autophagic vesicles containing engulfed organelles than untreated cells (Fig. 2d).

### OMT-induced autophagy protected SW982 cells from OMT-induced cytotoxicity

**Figure 3:**
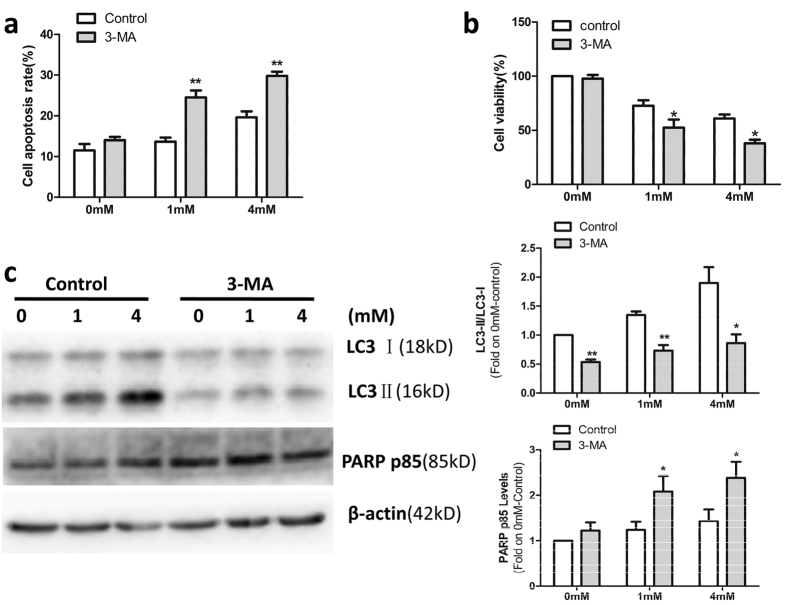
OMT-induced autophagy protected SW982 cells from OMT-induced cytotoxicity. SW982 cells were untreated or treated with 1 mM or 4 mM OMT for 48 h in the presence or absence of 5 mM 3-MA. The level of apoptosis was determined via an annexin-V-FITC/PI double staining assay (**a**). Cell viability was analyzed via MTT assay (**b**). The expression of LC3 and PARP p85 was detected by western blotting and relative densitometric analyses (**c**). Loading control was performed by evaluating β-actin expression in the same filter. The results are representative of 3 independent experiments and are expressed as the mean ± SD. The data were analyzed using Student’s t-test. *p < 0.05, **p < 0.01 compared with the control.

To determine the relationship between autophagy and OMT-induced cytotoxicity in SW982 cells, the autophagy inhibitor 3-MA was used in this experiment. As shown in Fig. 3c, a decrease in LC3-II expression was observed in SW982 cells treated with OMT in the presence of 3-MA which indicated that autophagy was inhibited. These results indicated that 3-MA increased the apoptosis (Fig. 3a) and decreased the viability of SW982 cells (Fig. 3b). Western blotting analysis of PARP cleavage confirmed these results. As shown in Fig. 3c, after 1 mM or 4 mM OMT treatment for 48 h, cleavage of PARP increased more dramatically in the presence of 3-MA.

**Figure 4:**
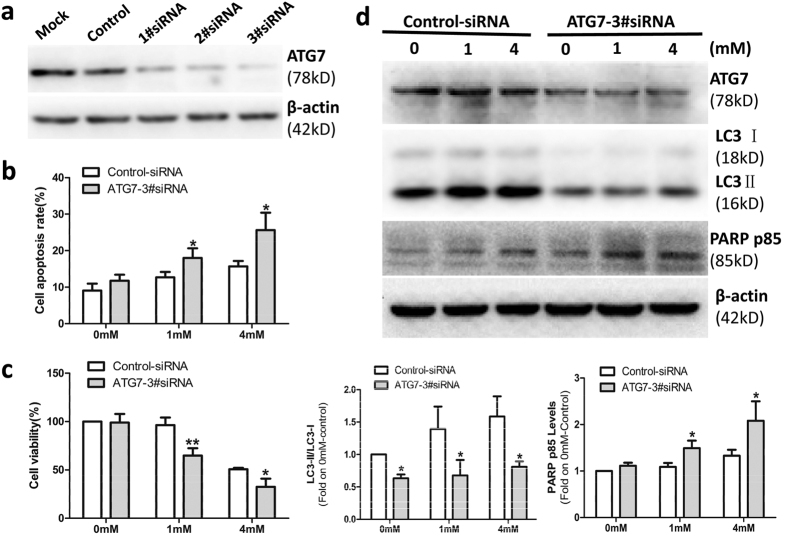
OMT-induced autophagy protected SW982 cells from OMT-induced cytotoxicity. Cells were transfected with Mock (Only transfection agent), Control-siRNA (80 nM), ATG7-1#siRNA(80 nM), ATG7-2#siRNA (80 nM) and ATG7-3#siRNA(40 nM 1#siRNA and 40 nM 2#siRNA) for 48 h, respectively, and the level of ATG7 protein was detected by western blotting (**a**). The cells were transfected with control or ATG7-3#siRNA, followed by 1 mM or 4 mM OMT treatment for 48 h. An annexin-V-FITC/PI double staining assay was used to determine the level of apoptosis (**b**), and an MTT assay was used to analyze cell viability (**c**). The expression of ATG7, LC3 and PARP p85 was detected by western blotting and relative densitometric analyses (**d**). Loading control was performed by evaluating β-actin expression in the same filter. *p < 0.05, **p < 0.01 compared with the control (0 mM or Control-siRNA). The *p* value was calculated using Student’s t-test. Data are presented as the mean ± SD of three independent experiments.

Because of concerns for the specificity of 3-MA as an autophagy inhibitor, we also examined the role of OMT-induced autophagy via knockdown of ATG7 gene. Figure 4a showed that ATG7 1#siRNA (80 nM), 2#siRNA (80 nM) and 3#siRNA (40 nM 1#siRNA and 40 nM 2#siRNA) significantly decreased the expression of ATG7. To confirm that the effects observed were due to the autophagy pathway rather than the off-target effects of the siRNA, ATG7 3#siRNA was used in the next experiments. As shown in Fig. 4, ATG7 3#siRNA increased the apoptosis (Fig. 4b and d) and decreased the viability of SW982 cells (Fig. 4c). Taken together, these results suggest that OMT-induced autophagy can protect SW982 cells from the cytotoxicity of OMT. Therefore, combining OMT with an autophagy inhibitor may be a novel strategy for the treatment of SS.

### HMGB1 modulated OMT-induced autophagy in SW982 cells

**Figure 5:**
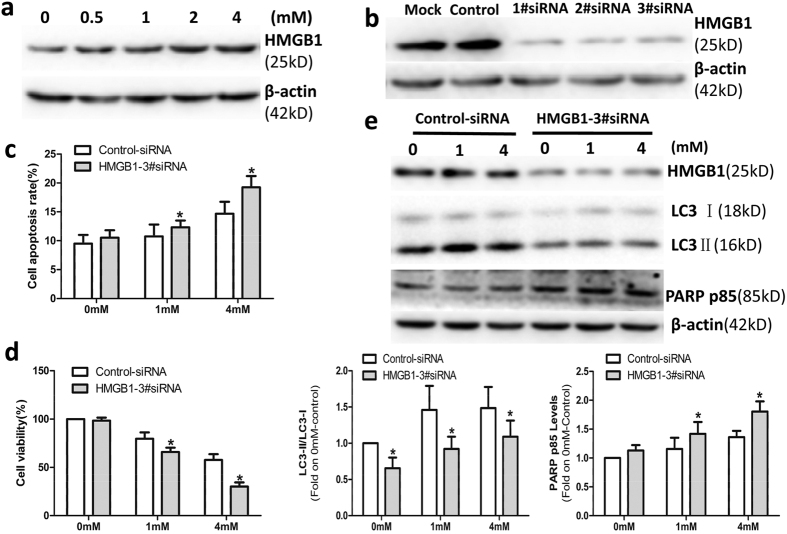
HMGB1 modulated OMT-induced autophagy in SW982 cells. Cells were treated with the indicated concentrations of OMT for 48 h, and the level of HMGB1 protein was detected by western blotting (**a**). SW982 cells were transfected with Mock (Only transfection agent), Control-siRNA (80 nM), HMGB1-1#siRNA(80 nM), HMGB1-2#siRNA (80 nM) and HMGB1-3#siRNA(40 nM 1#siRNA and 40 nM 2#siRNA) for 48 h, and the level of HMGB1 protein was detected by western blotting (**b**). The cells were transfected with control or HMGB1-3#siRNA, followed by 1 mM or 4 mM OMT treatment for 48 h. An annexin-V-FITC/PI double staining assay was used to determine the level of apoptosis (**c**), and an MTT assay was used to analyze cell viability (**d**). The expression of HMGB1, LC3 and PARP p85 was detected by western blotting and relative densitometric analyses (**e**). Loading control was performed by evaluating β-actin expression in the same filter. *p < 0.05 compared with the control (0 mM or Control-siRNA). The *p* value was calculated using Student’s t-test. Data are presented as the mean ± SD of three independent experiments.

Some studies have confirmed that HMGB1 is an important regulator of autophagy and that HMGB1-mediated autophagy promotes drug resistance to tumor therapy^31^,^33^,^34^. To investigate the relationship between HMGB1 and autophagy, we first detected the expression of HMGB1 in SW982 cells treated with OMT. SW982 cells were treated with OMT at concentrations of 0.5 mM, 1 mM, 2 mM or 4 mM for 48 h. As shown in Fig. 5a, OMT increased the expression of HMGB1in a dose-dependent manner, which indicated that OMT promoted the expression of HMGB1 in SW982 cells. We next investigated whether HMGB1-mediated autophagy was associated with this therapeutic effect. In this experiment, we used HMGB1 siRNA to decrease the expression of HMGB1. As shown in Fig. 5b, HMGB1 1#siRNA (80 nM), 2#siRNA (80 nM) and 3#siRNA (40 nM 1#siRNA and 40 nM 2#siRNA) significantly decreased the expression of HMGB1. To exclude the off-target effects of the siRNA, HMGB1 3#siRNA was used in the next experiments. Figure 5e showed that knockdown of HMGB1 via HMGB1 3#siRNA significantly decreased the expression of LC3-II, which indicated that OMT-induced autophagy may be regulated by HMGB1. Meanwhile, compared with the control group, HMGB1 3#siRNA significantly increased the cleavage of PARP (Fig. 5e) and apoptosis (Fig. 5c) and decreased the viability of SW982 cells (Fig. 5d). These results suggest that HMGB1 negatively modulates the sensitivity of SW982 cells to OMT and plays a vital role in the regulation of autophagy.

### OMT induced autophagy in SW982 cells through the HMGB1/Akt/mTOR pathway

**Figure 6:**
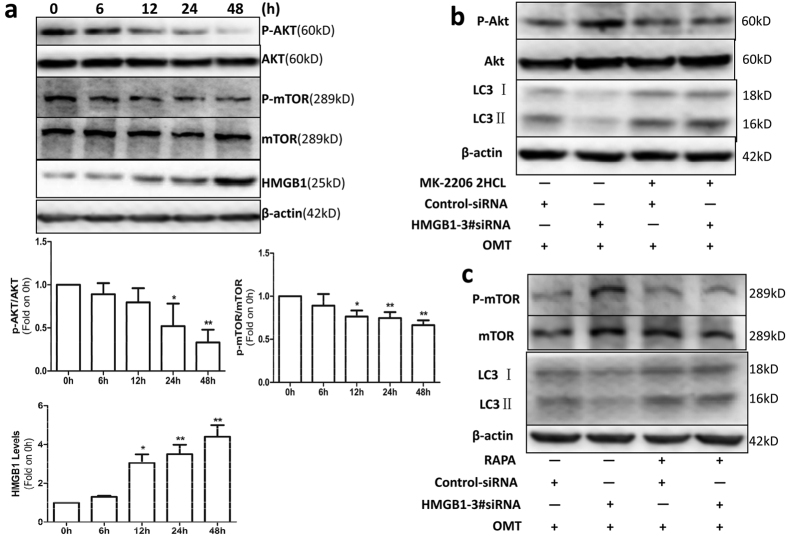
OMT induced autophagy in SW982 cells through the HMGB1/Akt/mTOR pathway. (**a**) Cells were treated with 2 mM OMT for the indicated times, and the protein expression of Akt, p-Akt, mTOR, p-mTOR and HMGB1 was detected by western blotting. (**b**) After SW982 cells were untreated or pretreated with 5 μM MK-2206 2HCL, an Akt inhibitor, for 2 h, they were transfected with HMGB1 3#siRNA or control siRNA, followed by 2 mM OMT treatment for another 48 h. Western blotting was used to determine the levels of LC3, Akt and p-Akt expression. The cells were untreated or pretreated with 100 nM Rapa for 2 h and then transfected with HMGB1 3#siRNA or control siRNA, followed by 2 mM OMT treatment for another 48 h. The expression of LC3, mTOR and p-mTOR was determined via western blotting (**c**). Loading control was performed by evaluating β-actin expression in the same filter. *p < 0.05, **p < 0.01 compared with the control (0 h). The *p* value was calculated using Student’s t-test. Data are presented as the mean ± SD of three independent experiments.

Because the Akt/mTOR pathway is a well-known autophagy-related signaling pathway, we investigated whether this pathway mediated OMT-induced autophagy and whether HMGB1 modulated autophagy through it in SW982 cells. Cells were treated with 2 mM OMT at times ranging from 6 h to 48 h, and Akt/mTOR pathway-related proteins and HMGB1 were detected by western blotting. As shown in Fig. 6a, the expression of p-Akt and p-mTOR was decreased, and the expression of HMGB1 was increased in a time-dependent manner, which indicated that OMT-induced autophagy may be mediated by the Akt/mTOR signaling pathway. We next analyzed the relationship between HMGB1 and the Akt/mTOR signaling pathway. After SW982 cells were treated with MK-2206 2HCL, an Akt inhibitor, the cells were then transfected with HMGB1 3#siRNA or control siRNA, followed by treatment with 2 mM OMT for 48 h. Figure 6b shows that HMGB1 3#siRNA increased the expression of p-Akt and decreased the expression of LC3-II, but did not decrease the expression of LC3-II, when Akt was inhibited by MK-2206 2HCL (Fig. 6b). Next, we investigated the effects of mTOR on HMGB1-regulated autophagy. We used an mTOR inhibitor, Rapa, to inhibit the expression of mTOR, knocked down HMGB1 expression with HMGB1 3#siRNA and then treated the cells with 2 mM OMT for 48 h. Similar to Akt, HMGB1 3#siRNA promoted the expression of p-mTOR and inhibited the expression of LC3-II; However, when Rapa was present, it was unable to regulate LC3-II expression (Fig. 6c). These data indicate that OMT-induced autophagy may be mediated by the HMGB1/Akt/mTOR signaling pathway.

## Materials and Methods

### Reagents

OMT was purchased from Chia Tai Tianqing Pharmaceutical Group Co., Ltd. (Lianyungang, China); its purity was over 98%, as determined via high-performance liquid chromatography. 3-(4, 5-dimethylthiazol-2-yl)-2, 5-diphenyltetrazolium bromide (MTT), rapamycin (Rapa), 3-methyladenosine (3-MA), bafilomycin A1(Baf A1) and antibodies to microtubule-associated protein 1 light chain 3 (anti-LC3) were obtained from Sigma-Aldrich (St. Louis, MO, USA). Anti-HMGB1, anti-cleaved-poly(ADP-ribose) polymerase (anti-cleaved-PARP), anti-Bax, anti-Bcl-2, anti-mammalian target of rapamycin (anti-mTOR) and anti-p-mTOR(phospho S2448) antibodies were obtained from Abcam (Cambridge, UK). Anti-ATG7, anti-p62, anti-Akt and anti-P-Akt (Ser473)antibodies were obtained from Cell Signaling Technology (Danvers, MA, USA). Anti-β-actin antibodies were purchased from Biosen (Beijing, China), E64d, pepstatin A and MK-2206 2HCL were obtained from Selleck (Shanghai, China).

### Cell culture and treatment

SW982 synovial sarcoma cells were obtained from the Shanghai Institute of Cell Biology, Chinese Academy of Sciences (Shanghai, China). The cells were grown in DME/F12 medium(Thermo Scientific, Waltham, MA, USA) supplemented with 10% fetal bovine serum (Gibco Life Technologies, USA), 100 units/mL penicillin and 100 μg/mL streptomycin at 37 °C under 5% CO_2_. The concentrations of OMT used in the present study ranged from 0.5 mM to 4 mM, and the culture periods ranged from 6 h to 48 h of continuous exposure to OMT; 5 mM 3-MA, 5 μM MK-2206 2HCL or 100 nM Rapa was used to pretreat the cells for 2 h prior to OMT treatment, and E64d/pepstatin A (10 μg/ml E64d and 10 μg/ml pepstatin A) or 100 nM Baf A1 was added to the cell cultures for the last 24 h of OMT treatment.

### MTT cell viability assay

An MTT assay was carried out to measure synovial sarcoma cell viability. The cells (1 × 10^4^ cells/well) were seeded in each well of a 96-well plate in 200 μl of culture medium, incubated overnight and then treated with OMT at indicated concentrations for 24, 48 and 72 h. A total of 20 μl of MTT solution (5 mg/mL) was added to each well, and the cells were incubated for another 4 h. Then, after the culture medium was discarded, the cells were lysed in 150 μl of dimethylsulfoxide (DMSO), and the optical density (OD) was measured at 550 nm with a microplate reader (Thermo, USA). The following formula was employed: cell viability(%) = (OD of the experimental sample/OD of the control group) × 100%.

### Cell apoptosis assay

An Annexin V-FITC Apoptosis Detection kit (Seven Sea PharmTech, China) was used to detect apoptosis following different treatments, according to the manufacturer’s instructions. Briefly, after various treatments, cells were harvested, washed twice with phosphate buffered saline (1 × PBS), and resuspended in 400 μl of annexin V binding buffer at a concentration of 4 × 10^5^ cells/ml. Then, the cells were incubated with fluorescein isothiocyanate (FITC)–annexin V (5 μl) for 15 minutes at room temperature in the dark and with propidium iodide (PI) (10 μl) for 5 minutes at 4 °C in the dark before being analyzed via flow cytometry (Millipore Corporation, Guava EasyCyte HT, CA, USA) to distinguish early apoptotic, late apoptotic, necrotic and viable cells from one another.

### Western blotting

Following different treatments, proteins were extracted with RIPA buffer (Beyotime, Shanghai, China) supplemented with protease inhibitor cocktail (Beyotime, Shanghai, China) on ice and then quantified using the BCA method (Thermo Scientific, Waltham, MA, USA). Forty microgram protein samples were separated using sodium dodecyl sulfate-polyacrylamide gel electrophoresis (SDS-PAGE) and then transferred to nitrocellulose membranes (Millipore, USA). The membranes were blocked in 10% skim milk in Tris-buffered saline–Tween-20 (TBST) for 2 hours at room temperature and then incubated with the following primary antibodies overnight at 4 °C: anti-PARP monoclonal antibody (1:1000), anti-p62 monoclonal antibody (1:1000), anti-ATG7 monoclonal antibody (1:1000), anti-Bcl-2 monoclonal antibody (1:1000), anti-Bax monoclonal antibody (1:1000), anti-β-actin monoclonal antibody (1:1000), anti-LC3 monoclonal antibody (1:1000), anti-HMGB1 monoclonal antibody (1:2000), anti-phosphomTOR (phospho S2448) monoclonal antibody (1:1000), anti-mTOR monoclonal antibody (1:1000), anti-phospho-Akt (Ser-473) monoclonal antibody (1:1000), and anti-Akt monoclonal antibody (1:1000). After three washes in TBST for 10 min each, the membranes were incubated with goat anti-rabbit horseradish peroxidase-conjugated secondary antibodies (1:10000) for 1 hour at room temperature. Then, the membranes were washed three times with TBST and detected using an electrochemiluminescence (ECL) system (GeneGnome 5, Synoptics Ltd, UK).

### Transmission electron microscopy

After SW982 cells were treated with OMT at indicated concentrations for 48 h, they were fixed with ice-cold 2% glutaraldehyde/0.1 M PBS (pH 7.2) and post-fixed in 1% osmium tetroxide. After being washed with PBS, the cells were dehydrated in a series of ascending ethanolconcentrations (30–100%), and embedded in propylene oxide/embedding resin (1:1). The resin blocks were cut into ultrathin sections with a LKB-V ultramicrotome (LKB, Sweden). Thin sections (60 nm) were placed on 200 mesh copper standard grids and stained with uranyl acetate and lead citrate. The ultrastructure of the cells was observed with a H-7650 transmission electron microscope (HITACHI, Ibaraki, Japan)^37^.

### Transfection experiments

Small interfering RNA (siRNA) targeting the HMGB1 cDNA sequence (1#siRNA: 5′-CAGGAGGAATACTGAACATTT-3′; 2#siRNA: 5′-GATGCAGCTTATACGAAATTT-3′), siRNA targeting the ATG7 cDNA sequence (1#siRNA: 5′-GCCGTGGAATTGATGGTATTT-3′; 2#siRNA: 5′-GGATCCTGGACTCTCTAAATT-3′) and a control siRNA cDNA sequence (5′-TTCTCCGAACGTGTCACGTTT-3′) were purchased from Gene Pharma Co. (Shanghai, China). SW982 cells were plated in 6- and 12-well plates at densities of 1.2 × 10^5^/well and 0.6 × 10^5^/well, respectively. Twenty-four hours after plating, HMGB1 siRNA or ATG7 siRNA or control siRNA at a concentration of 80 nM was transfected into the cells using the X-tremeGENE siRNA transfection agent (Roche), according to the manufacturer’s protocol. Four hours after transfection, the SW982 cells were treated as indicated and then used in the experiments.

### Statistical analysis

The results are presented as the mean ± standard deviation (SD). Comparisons between two groups were analyzed using Student’s t test or the Mann–Whitney U test for experiments in which the datasets were not normally distributed (GraphPad Prism 5.0 software; GraphPad Software, La Jolla, CA, USA). For all tests, *P* values less than 0.05 were considered statistically significant.

## Discussion

In the present study, we demonstrated that OMT inhibited cell viability and induced apoptosis and autophagy in SW982 cells. OMT-induced autophagy is an adaptive mechanism, and inhibition of autophagy enhanced OMT-induced cell apoptosis. In addition, we also found that autophagy regulated the sensitivity of SW982 cells to OMT, which may be mediated by the HMGB1/Akt/mTOR signaling pathway.

OMT is a type of alkaloid extracted from the traditional Chinese medicinal herb, *Sophora flavescens*. Although the antitumor activities of OMT have been observed in various tumors^18^,^19^,^20^,^21^,^22^,^23^,^24^,^38^,^39^, there are no reports regarding the effects of OMT on human synovial sarcoma. In this study, we found that OMT inhibited the viability of SW982 cells in a time- and dose-dependent manner. An annexin-V-FITC/PI double staining assay showed that OMT induced apoptosis in SW982 cells in a dose-dependent manner. Western blotting yielded the same result. Our results indicated that OMT inhibited cell viability and induced apoptosis in SW982 cells.

Although great progress has been made in the treatment of synovial sarcoma with chemotherapy, drug resistance has also become more common^9^,^10^. Some of the mechanisms underlying drug resistance in various diseases have been established, such as DNA repair mechanisms, drug export transporters and resistance to apoptosis^40^,^41^. Recent studies have found that autophagy is associated with drug resistance and that it protects cells from the cytotoxicity of chemotherapy drugs in many diseases, including cancer and inflammatory diseases^35^,^37^. In our study, we found that the expression of LC3-II was increased by OMT treatment. Transmission electron microscopy studies revealed that more autophagosomes were present in OMT treated cells than in non-treated cells. Increased LC3-II expression reflects only the number of autophagosomes formed, so autophagic flux should be detected^42^. We found that the expression of LC3-II was further increased in the presence of Baf A1 or E64d/pepstatin A via 2 mM OMT treatment in SW982 cells. These findings indicate that autophagy was induced by OMT in SW982 cells.

Autophagy is a cellular homeostasis mechanism that is essential for cell growth, differentiation, development and survival^43^. It is a survival strategy employed by cells experiencing nutrient deprivation or other stresses. To investigate the effects of autophagy induced by OMT in SW982 cells, 3-MA and ATG7 siRNA were used to inhibit autophagic activity. We found that viability of SW982 cells was decreased and that apoptosis and cleavage of PARP were dramatically increased after the autophagic activity was inhibited by 3-MA or ATG7 siRNA. These data suggest that OMT-induced autophagy protects SW982 cells from the cytotoxicity of OMT. Therefore, combining OMT and an autophagy inhibitor may be a novel strategy for the treatment of SS.

HMGB1, an important nuclear protein, is known to be involved in cancer development and is associated with drug resistance in tumor cells^31^,^44^,^45^,^46^. Recent studies have demonstrated that HMGB1 is an important regulator of autophagy and that HMGB1-regulated autophagy is a significant contributor to drug resistance following treatment with cytotoxic agents in tumor cells^32^,^34^,^47^,^48^. In the present study, we found that OMT increased the expression of HMGB1 in a dose- and time-dependent manner. After knockdown of HMGB1 by siRNA, the expression of LC3-II decreased significantly, which indicated that OMT-induced autophagy may be regulated by HMGB1. Furthermore, HMGB1 siRNA inhibited cell viability and promoted cell apoptosis in SW982 cells following OMT treatment. This evidence suggests that HMGB1 negatively modulated the sensitivity of SW982 cells to OMT and played a vital role in the regulation of autophagy.

The PI3K/Akt/mTOR signaling pathway is well known as an important regulatory pathway of autophagy. PI3K/Akt regulates autophagy mainly through modulation of mTOR activity. mTOR serves as a master regulator of autophagy^49^ and exists as two complexes, mTORC1 and mTORC2. mTORC1 negatively regulates autophagy by integrating upstream activating signals emitted by growth factors, amino acids, glucose, and energy through the PI3K/Akt pathway^50^. Thus, reduced mTOR activity induces autophagy. Yang *et al*.^51^ found that endogenous HMGB1 was an intrinsic regulator of autophagy in leukemia cells and that it enhanced leukemia cell chemoresistance, most likely through the PI3K/Akt/mTORC1 pathway. In the present study, we found that the expression of p-Akt and p-mTOR was decreased and that the expression of HMGB1 was increased in a time-dependent manner. Suppressing HMGB1 expression with HMGB1 siRNA increased the expression of p-Akt and p-mTOR and decreased the expression of LC3-II in SW982 cells treated with OMT, but did not decrease the expression of LC3-II, when an Akt inhibitor or mTOR inhibitor was present. These findings indicate that the HMGB1/Akt/mTOR signaling pathway may be involved in the process of OMT-induced autophagy.

In conclusion, our results reveal that OMT exhibits antitumor activity in SW982 cells and that OMT-induced autophagy protects cells from cytotoxicity. HMGB1 is increased after treatment with OMT and negatively modulates the sensitivity of SW982 cells to this drug. OMT-induced autophagy may be mediated by the Akt/mTOR pathway, and HMGB1 plays a vital role in the regulation of autophagy. Thus, combining OMT with an inhibitor of autophagy or HMGB1 may make OMT more effective in the treatment of SS. Further study is needed to confirm the therapeutic effects of OMT in SS and to elucidate the relationship between autophagy and apoptosis in *in vivo* experiments.

## Additional Information

**How to cite this article**: Cai, Y. *et al*. HMGB1-mediated autophagy decreases sensitivity to oxymatrine in SW982 human synovial sarcoma cells. *Sci. Rep.* **6**, 37845; doi: 10.1038/srep37845 (2016).

**Publisher's note:** Springer Nature remains neutral with regard to jurisdictional claims in published maps and institutional affiliations.

## Supplementary Material

Supplementary Figure
