# Supplementary material for: HMGB1-mediated autophagy decreases sensitivity to oxymatrine in SW982 human synovial sarcoma cells
Source: Sci Rep. 2016 Nov 29;6:37845. doi: 10.1038/srep37845 (PMC5126735; doi:10.1038/srep37845)

HMGB1-mediated autophagy decreases sensitivity to oxymatrine in SW982 human synovial sarcoma cells

Yongsong Cai[1], Peng Xu[2,*], Le Yang[2], Ke Xu[2], Jialin Zhu[2], Xiaoqing Wu[2], Congshan Jiang[3], Qiling Yuan[1], Bo Wang[4], Yuanbo Li[2] & Yusheng Qiu[1,*]

[1]Department of Orthopaedics of the First Affiliated Hospital, Xi'an Jiaotong University Health Science Center, Xi'an, 710061, China;

[2]Department of Joint Surgery, Xi'an Hong Hui Hospital, Xi'an Jiaotong University Health Science Center, Xi'an, 710054, China;

[3]Department of Biochemistry and Molecular Biology, School of Basic Medical Sciences, Xi'an Jiaotong University Health Science Center, Xi'an, 710061, China;

[4]Center for Translational Medicine, the First Affiliated Hospital of Xi'an Jiaotong University Health Science Center, Xi'an, 710061, China;

[*]Correspondence should be addressed to Peng Xu; sousou369@163.com; Yusheng Qiu; yusheng.qiu@mail.xjtu.edu.cn

Supplementary figure legend:

Supplementary Figure S1: Detection of autophagic flux. Cells were treated with 2 mM OMT for 48 h in the presence or absence of 100 nM Baf A1 (a) or E64d/pepstatin A(10μg/ml E64d and10μg/ml pepstatin A) (b). The expression of LC3 was detected by western blotting and relative densitometric analyses (a and b). Loading control was performed by evaluating β-actin expression in the same filter. The LC3-II/LC3-I ratios from 3 independent experiments are expressed as the mean± SD. The data were analyzed using Student's t-test. *p < 0.05, **p <0.01, ***p <0.001.

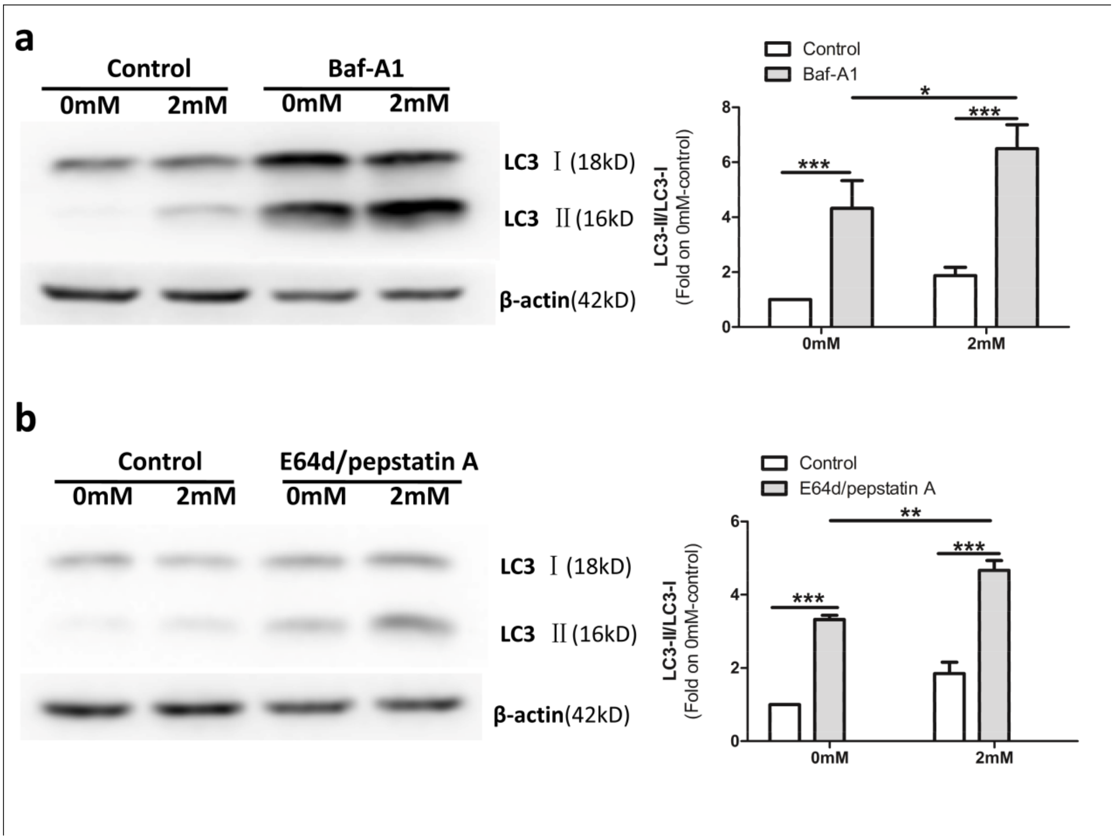

Supplementary Figure S2: The expression of p62 in SW982 cells treated with OMT. Cells were treated with the indicated concentrations of OMT for 48 h (a) or 2 mM OMT for the indicated times (b), and the expression of p62 was detected by western blotting and relative densitometric analyses (a and b). Loading control was performed by evaluating β-actin expression in the same filter. The results are representative of 3 independent experiments and are expressed as the mean± SD. The data were analyzed using Student's t-test. All $p > 0.05$, compared with the control (0 mM or 0 h).

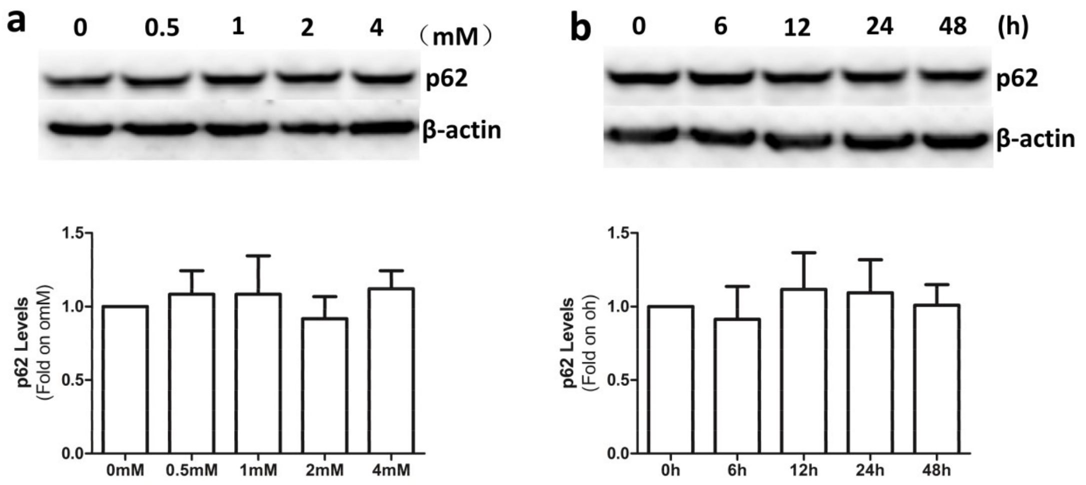

Supplementary Figure S3: Cells were treated with the indicated concentrations of OMT for 48 h (a) or 2 mM OMT for the indicated times (b), and the expression of LC3 was detected by western blotting (cells were seeded in 6-well plate and two wells of cells were treated with 4mM OMT for 48 h (a). In figure b, two wells of cells were treated for 48 h).

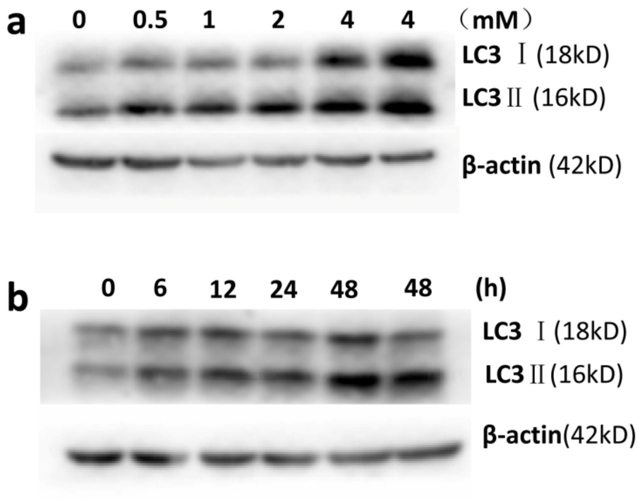

Supplement: Supplementary Figure [file srep37845-s1.pdf]
